# Exploring Different Incentive Structures Among US Adults Who Use e-Cigarettes to Optimize Retention in Longitudinal Web-Based Surveys: Case Study

**DOI:** 10.2196/49354

**Published:** 2023-12-13

**Authors:** Elizabeth Crespi, Johanna Heller, Jeffrey J Hardesty, Qinghua Nian, Joshua K Sinamo, Kevin Welding, Ryan David Kennedy, Joanna E Cohen

**Affiliations:** 1 Institute for Global Tobacco Control Department of Health, Behavior & Society Johns Hopkins Bloomberg School of Public Health Baltimore, MD United States

**Keywords:** incentive, conditional incentive, web-based survey, longitudinal study, follow-up, nicotine, e-cigarettes, tobacco, survey, retention, demographics, case study, optimization, adults

## Abstract

**Background:**

Longitudinal cohort studies are critical for understanding the evolution of health-influencing behaviors, such as e-cigarette use, over time. Optimizing follow-up rates in longitudinal studies is necessary for ensuring high-quality data with sufficient power for analyses. However, achieving high rates of follow-up in web-based longitudinal studies can be challenging, even when monetary incentives are provided.

**Objective:**

This study compares participant progress through a survey and demographics for 2 incentive structures (conditional and hybrid unconditional-conditional) among US adults using e-cigarettes to understand the optimal incentive structure.

**Methods:**

The data used in this study are from a web-based longitudinal cohort study (wave 4; July to September 2022) of US adults (aged 21 years or older) who use e-cigarettes ≥5 days per week. Participants (N=1804) invited to the follow-up survey (median completion time=16 minutes) were randomly assigned into 1 of 2 incentive structure groups (n=902 each): (1) conditional (US $30 gift code upon survey completion) and (2) hybrid unconditional-conditional (US $15 gift code prior to survey completion and US $15 gift code upon survey completion). Chi-square tests assessed group differences in participant progress through 5 sequential stages of the survey (started survey, completed screener, deemed eligible, completed survey, and deemed valid) and demographics.

**Results:**

Of the 902 participants invited to the follow-up survey in each group, a higher proportion of those in the conditional (662/902, 73.4%) than the hybrid (565/902, 62.6%) group started the survey (*P*<.001). Of those who started the survey, 643 (97.1%) participants in the conditional group and 548 (97%) participants in the hybrid group completed the screener (*P*=.89), which was used each wave to ensure participants remained eligible. Of those who completed the screener, 555 (86.3%) participants in the conditional group and 446 (81.4%) participants in the hybrid group were deemed eligible for the survey (*P*=.02). Of those eligible, 514 (92.6%) participants from the conditional group and 401 (89.9%) participants from the hybrid group completed the survey and were deemed valid after data review (*P*=.14). Overall, more valid completions were yielded from the conditional (514/902, 57%) than the hybrid group (401/902, 44.5%; *P*<.001). Among those who validly completed the survey, no significant differences were found by group for gender, income, race, ethnicity, region, e-cigarette use frequency, past 30-day cigarette use, or number of waves previously completed.

**Conclusions:**

Providing a US $30 gift code upon survey completion yielded higher rates of survey starts and completions than providing a US $15 gift code both before and after survey completion. These 2 methods yielded participants with similar demographics, suggesting that one approach is not superior in obtaining a balanced sample. Based on this case study, future web-based surveys examining US adults using e-cigarettes could consider providing the full incentive upon completion of the survey.

**International Registered Report Identifier (IRRID):**

RR2-10.2196/38732

## Introduction

Longitudinal studies allow researchers to monitor behaviors, understand dynamic relationships between different factors that influence behaviors, and establish causation by studying how behaviors evolve over time [1,2]. This is particularly important for rapidly evolving markets such as the e-cigarette market, where products and policies frequently change. However, obtaining a representative sample for longitudinal studies has grown more difficult, particularly as households have abandoned landlines [3]. The use of web-based surveys is increasing due to their cost-effectiveness, utility in quickly accessing large and diverse samples, and standardization of data collection processes, particularly in longitudinal studies [4,5].

High follow-up rates are critical in longitudinal research; missing data can significantly impact the validity and reliability of the findings [6,7]. Adequate follow-up is necessary to accurately track changes within study populations and reduce selective participation biases [8,9]. However, achieving high follow-up rates can be challenging, particularly in web-based surveys, where response rates are often lower than other methods [10]. Monetary incentives can be effective in increasing follow-up rates in web-based longitudinal research [11,12]. A 2006 study synthesizing existing research found that offering incentives increased the odds of individuals starting and completing a survey by 1.19 and 1.27 times, respectively [12]. However, the effectiveness of incentives may vary by incentive structure [13-20].

Incentive structures can be unconditional, granted regardless of survey participation, or conditional, awarded only upon survey completion [13]. In raising survey response rates, studies have found mixed results on the effectiveness of conditional versus unconditional incentives; while several (including mail-in, in-person, and web-based studies) found unconditional incentives more effective [13-17], 1 mail-in survey found no difference between conditional and unconditional incentives [18] and 1 web-based survey found conditional incentives more effective [19]. Additionally, 1 web-based survey found that conditional were more effective for higher incentive values and unconditional were more effective for lower incentive values [20]. Though most studies suggest that unconditional incentives are more effective in raising participation rates, conditional may be more cost-effective because incentives are provided only upon completion [15,18]. It should be noted that these studies vary in many regards that may affect responses to conditional versus unconditional incentives, such as study location, survey population, survey type (eg, mail-in and web-based), incentive type (eg, gift card and cash) and amount, and messaging regarding the survey invitation and incentive.

To our knowledge, no studies have examined a hybrid approach in which half of the incentive is unconditional and half is conditional upon valid survey completion. Additionally, there is a lack of research in incentive structures for web-based surveys of US adults who use e-cigarettes, a population that presents recruitment challenges due to lower prevalence. In this case study, we examine participant progress through a survey and resulting participant demographics for 2 incentive delivery structures among a sample of US adults who frequently use e-cigarettes to determine the most effective incentive structure for optimizing survey retention while maintaining representativeness.

## Methods

### Overview

The data used in this study are from follow-up participants in wave 4 of the Vaping and Patterns of e-Cigarette Use Research (VAPER) Study, a web-based longitudinal cohort study (July to September 2022) of US adults (aged 21 years or older) who use e-cigarettes ≥5 days per week. Participants were rescreened in each wave to ensure ≥5 days per week of e-cigarette use. More information about the study is available elsewhere [21]. In wave 4, participants who validly completed a survey in any prior waves (ie, waves 1, 2, 3, or any combination of these waves) and indicated interest in future surveys (N=1804) were randomly assigned into two groups (n=902 each), each offered a different incentive structure: (1) conditional (US $30 Amazon gift code upon survey completion) and (2) hybrid unconditional-conditional (US $15 Amazon gift code prior to survey completion and US $15 Amazon gift code upon survey completion). Participants were invited to the follow-up survey via an email and text containing a Research Electronic Data Capture (REDCap) survey link, information about the incentive structure, and, for the hybrid group, the initial US $15 gift code. Participants were reminded about the survey via up to 9 additional texts and emails over 9 weeks, each containing the same US $15 gift code. Upon survey completion (median completion time=16 minutes) and validation via data quality review, an email was sent with a US $30 (conditional group) or US $15 (hybrid group) gift code.

Participants’ progress was tracked to assess differences in participants passing through 5 distinct stages of the survey: starting the survey, completing a screener, being deemed eligible upon screener completion (e-cigarettes use ≥5 days per week), completing the full survey, and being deemed valid after a data quality review. Chi-square tests were used to assess differences between the two incentive groups in (1) participant completion of each survey stage and (2) participant demographics in the final sample (Stata 16.1; StataCorp LLC).

### Ethical Considerations

The Virginia Commonwealth University (approval number: HM20015004) and Johns Hopkins Bloomberg School of Public Health Institutional Review Boards (approval number: 9277) approved all study protocols. Participants provided informed consent. Study data used here were deidentified.

## Results

**Figure 1:**
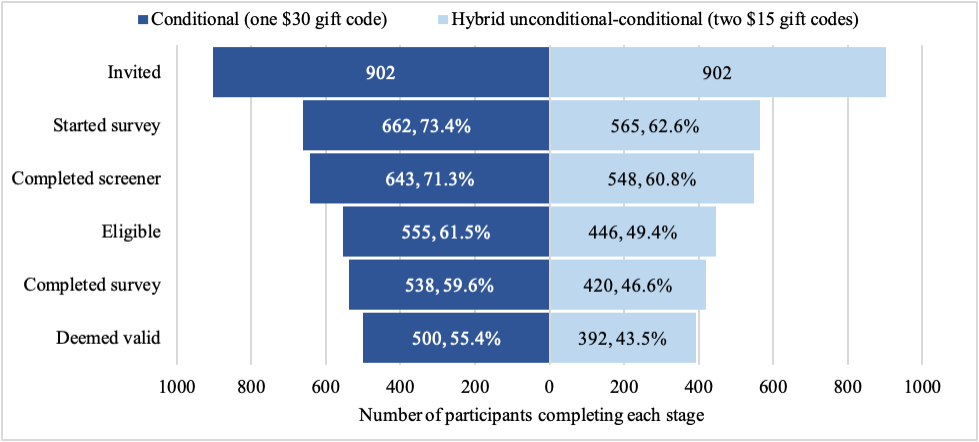
Number and percentage of participants completing each stage of a web-based survey by incentive structure in wave 4 of the Vaping and Patterns of e-Cigarette Use Research Study, a longitudinal survey of US adults who frequently use e-cigarettes (July to September 2022).

Of the 902 participants invited to the follow-up in each group, a higher proportion of those in the conditional (662/902, 73.4%) than the hybrid group (565/902, 62.6%) started the survey (*P*<.001). Of those who started the survey, 643 (97.1%) participants in the conditional and 548 (97%) participants in the hybrid group completed the screener (*P*=.89). Of those who completed the screener, 555 (86.3%) participants in the conditional and 446 (81.4%) participants in the hybrid group were deemed eligible for the survey (*P*=.02). Of those eligible, 538 (96.9%) participants from the conditional and 420 (94.2%) participants from the hybrid group completed the survey (*P*=.03). Of those who completed the survey, 500 (92.9%) participants from the conditional and 392 (93.3%) participants from the hybrid group were deemed valid after a data quality review and kept in our final sample (*P*=.81). Overall, more valid completions were yielded from the conditional (500/902, 55.4%) than the hybrid group (392/902, 43.5%; *P*<.001). Figure 1 depicts this information, with proportions displayed as a percentage of the total survey invitees rather than a percentage of those who completed the previous stage. Among those who validly completed the survey, no significant differences were found by group for gender, income, race, ethnicity, region, e-cigarette use frequency, past 30-day cigarette use, or number of waves previously completed (Table 1).

**Table 1:** Characteristics of participants deemed valid upon completion of the wave 4 follow-up survey of the Vaping and Patterns of e-Cigarette Use Research Study, a longitudinal web-based survey of US adults who frequently use e-cigarettes from July to September 2022, by incentive structure group.

Characteristics		Conditional (n=500), n (%)	Hybrid unconditional-conditional (n=392), n (%)	Total (N=892)	*P* value	
**Sex**						.33
	Male	211 (42)	154 (39)	365		
	Female	278 (56)	234 (60)	512		
	Nonmale or female	10 (2)	3 (1)	13		
	Prefer not to answer	1 (0)	1 (0)	2		
**Age**						.73
	21-29 years	165 (33)	125 (32)	290		
	30 years or older	335 (67)	267 (68)	602		
**Income**						.83
	<US $40,000	226 (45)	182 (46)	408		
	≥US $40,000	261 (52)	202 (52)	463		
	Prefer not to answer	13 (3)	8 (2)	21		
**Race**						.13
	Black	16 (3)	8 (2)	24		
	Multiracial	68 (14)	48 (12)	116		
	Non-Black or White	16 (3)	23 (6)	39		
	White	392 (78)	301 (77)	693		
	Prefer not to answer	8 (2)	12 (3)	20		
**Ethnicity**						.67
	Non-Hispanic or Latino	446 (89)	348 (89)	794		
	Hispanic or Latino	43 (9)	38 (10)	81		
	Prefer not to answer	11 (2)	6 (2)	17		
**Region**						.16
	Northeast	66 (13)	47 (12)	113		
	Midwest	113 (23)	70 (18)	183		
	South	194 (39)	153 (39)	347		
	West	127 (25)	122 (31)	249		
**e-Cigarette use frequency**						.93
	Daily	479 (96)	376 (96)	855		
	Nondaily (5 or 6 days per week)	21 (4)	16 (4)	37		
**Smoking status**						.25
	Currently smoking (smoked ≥100 cigarettes in lifetime and at least once in the past 30 days)	127 (25)	86 (22)	213		
	Quit or recently stopped smoking (smoked ≥100 cigarettes in lifetime and not in the past 30 days)	299 (60)	234 (60)	533		
	Never smoked (smoked <100 cigarettes in lifetime)	74 (15)	72 (18)	146		
**Total number of waves completed**						.15
	2	179 (36)	144 (37)	323		
	3	154 (31)	99 (25)	253		
	4	167 (33)	149 (38)	316		

## Discussion

### Principal Findings

Understanding how to optimize follow-up rates can help researchers design more robust studies and improve data quality. In this case study of adults using e-cigarettes in the fourth wave of an ongoing longitudinal survey, we assessed differences in survey initiation and completion and participant demographics for 2 incentive structures; the conditional structure (US $30 gift code upon survey completion) yielded more survey starts and completions than the hybrid structure (US $15 gift code both before and after survey completion). These findings add to the literature by providing evidence in favor of the conditional structure, consistent with some previous work [19], but inconsistent with other studies that support the effectiveness of the unconditional structure [13-17]. Further consideration is needed to understand why literature regarding conditional versus unconditional incentives is mixed; this may relate to other features that vary across studies (eg, study location, sample population, when data collection occurred, survey modality, incentive amounts and delivery methods, and messaging about the survey and incentive). Additional research is needed to understand how these factors affect survey participation rates and if other approaches may be effective in improving response rates and recruitment cost-effectiveness (eg, different breakdown of pre- or postsurvey incentive amounts, different incentive formats like cash or charity donations, additional incentives for earlier responders, and more personalized approaches to build rapport).

Additionally, we find that the conditional and hybrid approaches yielded participants with similar demographics, suggesting that for this case study, one approach was not superior to the other in maintaining the representativeness of the sample. A 2014 study examining participation rates in a web-based questionnaire similarly found no differences by age or gender between conditional and unconditional incentives [15]. Other studies have suggested that the response to conditional versus unconditional incentives may vary by geographic location [22]. Thus, the results outlined in this case study may not be generalizable to other populations or locations. Future studies examining various incentive structures and methods for optimizing follow-up rates should consider if the structures or methods may differentially impact response rates for certain demographics or geographic locations.

### Strengths and Limitations

The strengths of this work include the large sample size and the randomized assignment of participants to each group. It should be noted that these results are intended as a case study of US adults who use e-cigarettes 5 or more days per week; results may not be generalizable to other populations within or outside of the United States. Additionally, in previous VAPER Study waves, all participants received conditional incentives; it is possible that the transition from conditional to hybrid incentives may have influenced survey initiations and completions.

### Conclusions

In our study, participants receiving a US $30 gift code upon completion of the survey were more likely to complete the survey than those receiving a US $15 gift code both before and after completing the survey; participant demographics in the final sample for these 2 approaches did not differ significantly. Future web-based surveys, particularly those examining US adults who use e-cigarettes, could consider providing the full incentive upon survey completion. Future research should continue to examine approaches for increasing follow-up rates in web-based longitudinal surveys and consider how these approaches may affect response rates across varying populations.

## Abbreviations

REDCap: Research Electronic Data Capture
VAPER: Vaping and Patterns of e-Cigarette Use Research

## Data Availability

Interested investigators may contact the corresponding author for more information on accessing these data.
